# Single-cell profiling of peripheral blood mononuclear cells from patients treated with oncolytic adenovirus TILT-123 reveals baseline immune status as a predictor of therapy outcomes

**DOI:** 10.1038/s41417-025-00901-z

**Published:** 2025-04-10

**Authors:** Tatiana V. Kudling, Dmitrii Bychkov, James H. A. Clubb, Santeri A. Pakola, Victor Arias, Elise Jirovec, Mirte van der Heijden, Nea Ojala, Dafne C. A. Quixabeira, Lyna Haybout, Katriina J. Jalkanen, Tuomo Alanko, Riikka Havunen, Suvi Sorsa, Claudia Kistler, Anna Kanerva, Otto Hemminki, Joao M. Santos, Victor Cervera-Carrascon, Akseli Hemminki

**Affiliations:** 1 Cancer Gene Therapy Group, Translational Immunology Research Program, Faculty of Medicine, University of Helsinki, Helsinki, Finland; 2 Digital Diagnostics Group, Institute for Molecular Medicine Finland (FIMM), Institute of Life Science (HiLIFE), University of Helsinki, Helsinki, Finland; 3 TILT Biotherapeutics Ltd, Helsinki, Finland; 4 Comprehensive Cancer Center, Helsinki University Hospital (HUS), Helsinki, Finland; 5 Docrates Cancer Center, Helsinki, Finland; 6 Department of Obstetrics and Gynecology, Helsinki University Hospital and University of Helsinki, Helsinki, Finland; 7 Department of Urology, Helsinki University Hospital, Helsinki, Finland

**Keywords:** Biomarkers, Cancer immunotherapy

## Abstract

Oncolytic adenovirus Ad5/3-E2F-d24-hTNFa-IRES-hIL2 (TILT-123, igrelimogene litadenorepvec) shows promise as a therapeutic agent capable of causing tumor regression and activating host immunity. A phase I clinical study TUNIMO (NCT04695327) assessed its safety as monotherapy in patients with various solid tumors. Through single-cell profiling of peripheral blood, we identified distinct immunological features distinguishing responders from non-responders. Specifically, at baseline, responders demonstrated enhanced cytotoxic markers and stronger immune cell communication networks. Moreover, higher baseline CD16+ monocytes correlated with improved survival, while elevated regulatory T cells predicted poor response. T and B cell evaluation revealed contrasting patterns: responders showed higher numbers of T cells with predicted specificity to both adenovirus and tumor antigens, while elevated total memory B cells, regardless of specificity, predicted poor survival. Several T and B cell receptor segments matched those previously reported in other viral infections, suggesting possible cross-reactive immune responses. These findings emphasize that comprehensive biomarker analysis of peripheral blood should include not only cell frequencies but also transcriptional changes and distinct patterns of cellular and humoral immunity.

## Background

Engineered oncolytic viruses (OVs) offer a promising anti-cancer approach by precisely targeting and killing tumor cells, while activating the host immune system. Upon infection, OVs are able to deliver transgenes to the tumor site allowing their high expression and thus priming immune cells, including T cells [1]. In that regard, tumor necrosis factor α (TNFα) and interleukin 2 (IL2) have been identified as highly potent trafficking and activation cytokines in the context of T cells [2]. We have previously constructed an oncolytic adenovirus coding for human TNFα and IL-2, Ad5/3-E2F-d24-hTNFα-IRES-hIL2 (TILT-123 or igrelimogene litadenorepvec) allowing selective replication in cancer cells and high local expression of these transgenes [3]. TILT-123 was shown safe in preclinical safety studies [4] and demonstrated promising anti-tumor efficacy both as monotherapy and in combination with immune checkpoint inhibitors or adoptive tumor-infiltrating lymphocyte therapies across various solid cancers, including ovarian cancer, melanoma and head and neck cancers [5–7]. Notably, TILT-123 effectively reduces tumor growth both through local and intravenous administration [8]. The ex vivo analysis of primary human histocultures of various origin treated with TILT-123 also revealed enhanced cell killing and a significant increase in activated cytotoxic T cells along with the upregulated expression of immunostimulatory molecules [9, 10].

Following several years of pre-clinical work, a phase I trial TUNIMO (NCT04695327) was initiated. This open-label, dose-escalation trial was designed to evaluate, as primary endpoint, the safety of TILT-123 as monotherapy in patients with advanced solid tumors. An initial dose was administered intravenously, followed by multiple intratumoral injections. Collected clinical data indicate that TILT-123 is safe, well-tolerated with promising signs of efficacy in treating both injected and non-injected lesions in these heavily pre-treated patients [11]. However, despite overall promising efficacy data in some patients, not all patients showed measurable responses after the therapy. This variation in treatment benefit can probably be attributed to a range of factors including tumor origin, genetic variability, and immune system status [12]. There is a critical need for reliable predictive and prognostic biomarkers that would enable identification of patients most likely to benefit from TILT-123.

Single-cell profiling has increasingly become a valuable tool in preclinical and translational cancer research, enabling detailed characterization of heterogeneous cell populations of both malignant and non-malignant origin. However, despite its extensive application, only a few studies address clinical trials of OVs using this approach [13, 14]. Ramelyte et al. studied cellular responses to talimogene laherparepvec (T-VEC) in patients with cutaneous B-cell lymphoma. Rapid activation and intratumoral infiltration of natural killer cells, monocytes, and dendritic cells was observed as well as enrichment in cytotoxic T cells, although no direct correlations with clinical efficacy were established [13]. Zhang et al. focused on the effects of intraperitoneally administered oncolytic adenovirus H101 on malignant ascites in a cohort of patients with several tumor indications, including pancreatic, ovarian, gastrointestinal and biliary cancers [14]. Responding and non-responding patients were compared and those with the upregulated immune signaling pathways in tumor cells and a higher proportion of CLEC10A^+^ dendritic cells and cytotoxic CD8^+^ T cells at baseline benefited from the therapy.

The research presented in this study utilizes the peripheral blood mononuclear cells (PBMCs) from TUNIMO trial with the aim of identifying potential systemic biomarkers of treatment benefit. Our data provide a comprehensive understanding of the patient’s immune status before and after treatment with TILT-123, significantly contributing to the development of next-generation trials for currently incurable cancers.

## Methods

### Patients

Out of a total 20 patients treated in TUNIMO, 9 patients were imaged with PET scans and diagnostic CT scans on day 78 after treatment initiation and were available for single-cell profiling (Table 1). The included patients had following cancer types: sarcomas (*n* = 3), non-small cell lung cancer (*n* = 1), melanoma (*n* = 1), ovarian cancer (*n* = 1), thyroid cancer (*n* = 1), head and neck cancers (*n* = 2). Eligibility criteria were published previously [11].

**Table 1:** Patient characteristics and responses.

Patient ID	Tumor type	Cohort/Virus dose	Stage	No. of prior systemic treatments	PET criteria	Tumor size change based on PET, %	Overall response	Overall survival, days
20103	Anaplastic thyroid carcinoma	3/ 3 × 10^11^ VP (IV) + 1 × 10^11^ VP (IT)	IV	1	PMR	−100.00	Responder	983^a^
20108	Adenoid cystic carcinoma	5/ 2 × 10^12^ VP (IV) + 3 × 10^11^ VP (IT)	IV	0	SMD	221.00	Responder	221
20202	NSCLC	1/ 3 × 10^9^ VP (IV) + 3 × 10^9^ VP (IT)	IV	5	PMR	−44.47	Responder	102
20204	Myxoid liposarcoma	2/ 3 × 10^10^ VP (IV) + 3 × 10^10^ VP (IT)	IV	5	SMD	18.03	Responder	821
20206	High grade serous ovarian carcinoma	2/ 3 × 10^10^ VP (IV) + 3 × 10^10^ VP (IT)	IIIC	16	SMD	−2.69	Responder	192
20211	Cutaneous melanoma	3/ 3 × 10^11^ VP (IV) + 1 × 10^11^ VP (IT)	IV	3	PMD	−5.61	Non-Responder	377
20212	Leiomyosarcoma	4/ 1 × 10^12^ VP (IV) + 3 × 10^11^ VP (IT)	IV	4	PMD	27.90	Non-Responder	557
20217	Myxoid liposarcoma	6/ 4 × 10^12^ VP (IV) + 5 × 10^11^ VP (IT)	IV	4	PMD	127.27	Non-Responder	131
20219	High grade mucoepidermoid carcinoma of the parotid gland	6/ 4 × 10^12^ VP (IV) + 5 × 10^11^ VP (IT)	IV	2	PMD	222.93	Non-Responder	239

*NSCLC* non-small cell lung cancer, *PMD* progressive metabolic disease, *SMD* stable metabolic disease, *PMR* partial metabolic response.

^a^Patient alive at data-cutoff.

### Treatments

The patients were administered with multiple doses of TILT-123 beginning with an intravenous dose on day 1, and followed by intratumoral doses on days 8, 22, 36, 48, and 64. Patients who potentially benefited from the treatment were eligible to continue in the extension protocol and receive further doses of TILT-123 beyond the primary endpoint. The intravenous dose was escalated between 3 × 10^9^ and 4 × 10^12^ viral particles (VP) while the intratumoral doses were escalated from 3 × 10^9^ to 5 × 10^11^ VPs between the six cohorts according to the dose-escalation scheme.

### Antitumor efficacy and survival

Antitumor efficacy was evaluated on day 78 using PET scans with ^18^F-FDG. Tumor responses were assessed using PET-based criteria, detailed in Supplementary Table S1. Patients who met the criteria for stable metabolic disease or better, according to PET-based criteria, were considered Responders in this study. Survival data were obtained from the electronic clinical trial system, with a data cutoff specified as 01/09/2024.

### PBMCs isolation

PBMCs were isolated from patients’ whole blood using BD Vacutainer CPT Mononuclear Cell Preparation Tubes (BD Bioscience, NJ, USA) in accordance to the manufacturer’s instruction. PBMCs were isolated within 3 h after blood collection and stored in the freezing media (10% dimethyl sulfoxide (DMSO), 90% fetal bovine serum (FBS)) at −140 °C until further use.

### Single-cell suspension preparation

Frozen vials of PBMCs, collected on days −14, and 64 were rapidly thawed in a 37 °C water bath and mixed with pre-warmed RPMI media with 10% FBS and centrifuged at 200 × *g* for 10 min at +4 °C. The cell pellet was resuspended in 0.5 mL of pre-warmed RPMI with 10% FBS and cell density was measured. Prior capturing, 5 × 10^5^ cells from each sample were labeled with Single-Cell Multiplexing Kit (BD Biosciences, NJ, US), pooled together, and stained with Calcein AM (BD Biosciences, NJ, US) and DRAQ7 (BD Biosciences, NJ, US) to determine live cell numbers with the BD Rhapsody Scanner (BD Biosciences, NJ, US).

### Single-cell RNA sequencing

Single cell separation and library preparation was performed using the microwell-based BD Rhapsody scRNA-Seq platform with the BD Rhapsody Human Immune Response Targeted amplification kit (BD Biosciences, NJ, US) and BD Rhapsody TCR/BCR Full length amplification kit (BD Biosciences, NJ, US) according to the manufacturer’s instructions. Briefly, 1 × 10^4^ PBMCs per sample were loaded on the BD Rhapsody cartridge for single cell separation. cDNA was amplified using cDNA kit (BD Biosciences, NJ, US). Subsequently, libraries for mRNA transcripts, sample tags and TCR/BCR were prepared according to the manufacturer’s instructions. TCR/BCR libraries were sequenced on the NovaSeq 6000 device (Illumina, CA, US) to generate 250 bp paired-end reads, and mRNA and sample tag libraries were sequenced on the NovaSeq X Plus device (Illumina, CA, US) to generate 150 bp paired-end reads.

Resulting FASTQ files were processed using the BD Rhapsody Sequence Analysis Pipeline run (Seven Bridges, IL, US), and loaded into the R package Seurat (v 5.0.0) [15] for quality control and downstream analyses.

### Data processing

Quality control (QC) measurements for each sample are provided in Supplementary Table S2. We obtained 94,405 putative cell counts from 18 PBMCs samples (before and after the treatment for each patient) and used the following QC procedure: (1) select sample tag positive cells, (2) exclude doublets, (3) select cells that had more than 200 transcripts per cell, (4) select cells for which the number of genes was in range of 300 and 5000. Unsupervised clustering with dimension reduction analysis was performed by the Seurat with a clustering resolution of 0.6. This analysis included normalization, principal component analysis, integration, clustering and dimensionality reduction with uniform manifold approximation and projection (UMAP), and differential expression analysis of clusters.

### Cell type annotation

Highly variable genes (HVGs) were obtained using the FindVariableFeatures() function with default parameters. The top 2000 HVGs, which were decreasingly ordered based on dispersion, were selected as input to downstream analysis. Data were scaled using ScaleData(), integrated using IntegrateData() and dimension reduction was performed using RunPCA() and RunUMAP(dims=1:10) functions. The top 20 principal components (PCs) were used to construct nearest-neighbor graphs, and identify cell clusters using the FindNeighbors() and FindClusters() functions of the Seurat package. 15 major cell types were identified manually using following markers: Classic Monocytes (*CD14*^*+*^), CD16^+^ Monocytes (*FCGR3A*^*+*^), Naïve CD4 T cells (*CD3E*^*+*^*CD4*^*+*^*IL7R*^*+*^*CCR7*^*+*^), Central Memory CD4+ T cells (*CD3E*^*+*^*CD4*^*+*^*IL7R*^*+*^*CCR7*^*+*^*CD44*^*+*^*CD28*^*+*^*FAS*^+^), Central Memory CD8+ T cells (*CD3E*^*+*^*CD8B*^*+*^*IL7R*^*+*^*CCR7*^*+*^*FAS*^+^), Effector Memory CD8+ T cells (*CD3E*^*+*^*CD8B*^*+*^*CCR7*^*−*^*CD28*^*+*^*FAS*^*+*^), Effector CD8^+^ T cells (*CD3E*^*+*^*CD8B*^*+*^*GNLY*^*+*^), CD56^dim^ NK cells (*CD3E*^*-*^ *NCAM*^*dim*^*GNLY*^*+*^*NKG7*^*+*^), CD56^bright^ NK cells (*CD3E*^*−*^*NCAM*^*bright*^), NK-like T cells (*CD3E*^*+*^*KLRB1*^*+*^), T regulatory cells (*CD3E*^*+*^*CD4*^*+*^*FOXP3*^*+*^), B cells (*MS4A1*^*+*^), Plasma cells (*MS4A1*^*−*^*JCHAIN*^*+*^), plasmocytoid Dendritic Cells (pDC) (*CD4*^*+*^*FCER1A*^*+*^*IL3RA*^*+*^), conventional Dendritic Cells (cDC) (*FCER1A*^*+*^*CD1C*^*+*^).

### Differential expression analysis and pathway analysis

FindMarkers() function in Seurat was used to perform differential gene expression analysis between different groups and time points using Wilcoxon Rank Sum test with Benjamini-Hochberg method for multiple testing correction. Differentially expressed genes (DEG) were filtered to retain those with fold change (avg_log2FC) ≥ 0.25 and adjusted *p* value (p_val_adj) *≤*0.05. KEGG pathway enrichment analysis was performed using enrichKEGG() function in R package clusterProfiler (v.4.12.0) [16, 17]. Cell-cell interaction analysis was performed using R package CellChat (v.1.5.0) [18, 19]

### BCR and TCR clonotypes characterization

scRepertoire (v.2.0.4) package [20] was used to evaluate T- and B-cell clonotypes diversity, similarity, and rearrangement. A deep learning-based method ERGO II [21, 22] was used for the prediction of anti-adenoviral specificity of T cells. Autoencoder based model with McPAS training database was used to calculate prediction scores, while V and J genes, TCRα and T cell type were used for more accurate predictions.

The list of potential adenoviral epitopes was obtained from IEDB [23] with the following settings: (1) Epitope: linear peptide; (2) Epitope source: Human adenovirus 5; (3) Host: Human; (4) Assay: T cell; (5) MHC restriction: Any; (6) Disease: Any.

The list of potential tumor-associated epitopes was obtained from CEDAR [24] with the following settings: (1) Epitope: linear peptide; (2) Epitope source: Neoantigen/Germline/Self/Host antigen; (3) Host: Human; (4) Assay: T cell; (5) MHC restriction: Any; (6) Disease: Cancer Stage IV, Metastatic.

Selected peptides (Supplementary Table S3) were used to find a best matching pair for each captured and sequenced TCRαβ. The chains with prediction score of more than 0.97 were selected for further analysis.

### Neutralizing antibody analysis

Neutralizing antibodies (NAb) from complement-inactivated serum samples were determined using the DualLuciferase® Reporter Assay System (Promega, WI, USA), as described previously [25]. The NAb titer was determined to have the lowest dilution of the serum that blocked at least 80% of luciferase activity.

### Statistics

All statistical calculations were performed by GraphPad Prism 9.2.1 (GraphPad Software, MA, US) or RStudio (4.3.0). The normality of the data was assessed using Shapiro–Wilk test, and the equality of variances—using Levene’s test. Unpaired *t*-test was used for group comparison when data was normally distributed, while non-parametric Wilcoxon test was used to compare non-normally distributed data. Paired tests were used for timepoint comparisons of the groups. Correlations between variables were investigated either using Pearson correlation or Spearman’s rank correlation coefficient based on the data distribution. For cell population comparisons, given the exploratory nature of this phase I trial and small sample size, multiple testing corrections were not applied (statistical approaches for differential gene expression analysis are detailed in the respective Methods section). Results with *p* value of less than 0.05 were considered statistically significant.

## Results

### Group comparison of cell populations revealed the proportion of CD16 monocytes and Tregs at baseline as associated with response and survival

Cancer patients with different tumor indications were enrolled in the TILT-123 monotherapy clinical study. Clinical information including the response to the therapy, previous treatments and overall survival were published previously [11]. The patients were divided to Responders and Non-Responders, based on PET-criteria (Table 1). However, our analysis also examines outcomes based on survival length (>12 months vs. <12 months) as an independent metrics. These two classifications (response and survival) don’t always align, as some Non-Responders experienced longer survival. This discrepancy may be partly explained by limitations of PET imaging in immunotherapy evaluation, where activated T cells can increase glucose uptake creating potentially misleading signals [26]. We include both metrics as they provide complementary insights into different aspects of clinical benefit, with metabolic response reflecting early treatment effects and survival capturing long-term outcomes.

The total number of PBMCs captured for single-cell RNA sequencing 14 days prior to the treatment with TILT-123 (BL, baseline), and after the treatment on day 64 (D64) reached nearly 124,000 cells with the mean of approximately 4000 cells per sample, mean coverage of 20,700 reads/cell and nearly 1000 genes/cell (Supplementary Table S2).

**Fig. 1:**
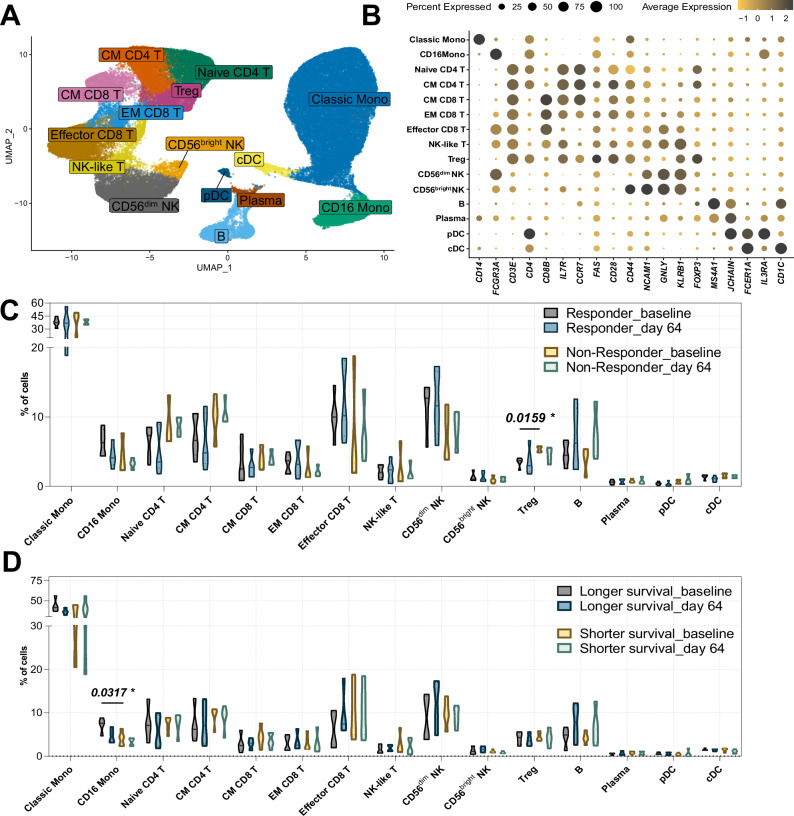
Cell clustering and cell dynamics in patients underwent TILT-123 therapy. **A** UMAP plot of combined baseline and day 64 PBMCs collected from patients before (BL) and after (D64) they were administered with TILT-123. CM CD4 T = Central Memory CD4 T cells, CM CD8 T = Central Memory CD8 T cells, EM CD8 T = Effector Memory CD8 T cells, Treg = T regulatory cells, cDC = conventional dendritic cells, pDC = plasmacytoid dendritic cells. **B** Dot plot showing expression of common markers used for clustering annotation. **C** Cell type frequencies in individual patients at baseline (BL) and after the treatment (D64) grouped based on the treatment response (Responders *n* = 5, Non-Responders *n* = 4). Data are shown as violin plots displaying distribution density with median indicated by the central line. Values are expressed as percentage of total cells per sample. **D** Cell type frequencies in individual patients at baseline (BL) and after the treatment (D64) grouped based on survival length (Longer survivors *n* = 4, Shorter survivors *n* = 5). Patients with OS > 12 months were considered Longer survivors. Data are shown as violin plots with median values indicated. Differences between groups (Responders vs. Non-Responders, Longer vs. Shorter survivors) were assessed using unpaired *t*-test or Wilcoxon test depending on the data distribution. For comparing baseline and day 64 timepoints within groups, paired *t*-test or Wilcoxon signed-rank test were used. *p* values < 0.05 were considered significant.

Analysis of sequencing data showed high consistency of the samples and enabled detection and annotation of distinctive clusters of immune cells based on the commonly used cell-type markers (Fig. 1A, B, individual UMAP plot are shown in Supplementary Fig. S1). Overall, monocytes made up the majority of the cells with the mean of 43%, followed by 37.7% T cells, 11% NK cells and 5.7% B cells (Supplementary Table S4). Comparison between response groups showed that most cell types had no statistical significance, although cytotoxic CD56^dim^ NK cells and Effector CD8 T cells demonstrated a trend toward higher levels in Responders, while Naïve and Central Memory CD4 T cells showed a trend toward higher levels in Non-Responders. The only significant difference was observed in Tregs, showing higher percentage in Non-Responders at baseline (*p* = 0.0159). Both groups showed increased B cell numbers at day 64 compared to baseline (Fig. 1C). Analysis based on survival (patients who survived longer that 1 year were considered long survivors in this study) showed similar pattern with most cell populations showing no significant differences, except CD16+ monocytes being significantly higher in patients with longer survival (*p* = 0.0317). Similar to response comparison, B cells showed tendency to increase by day 64 regardless of survival status (Fig. 1D).

**Fig. 2:**
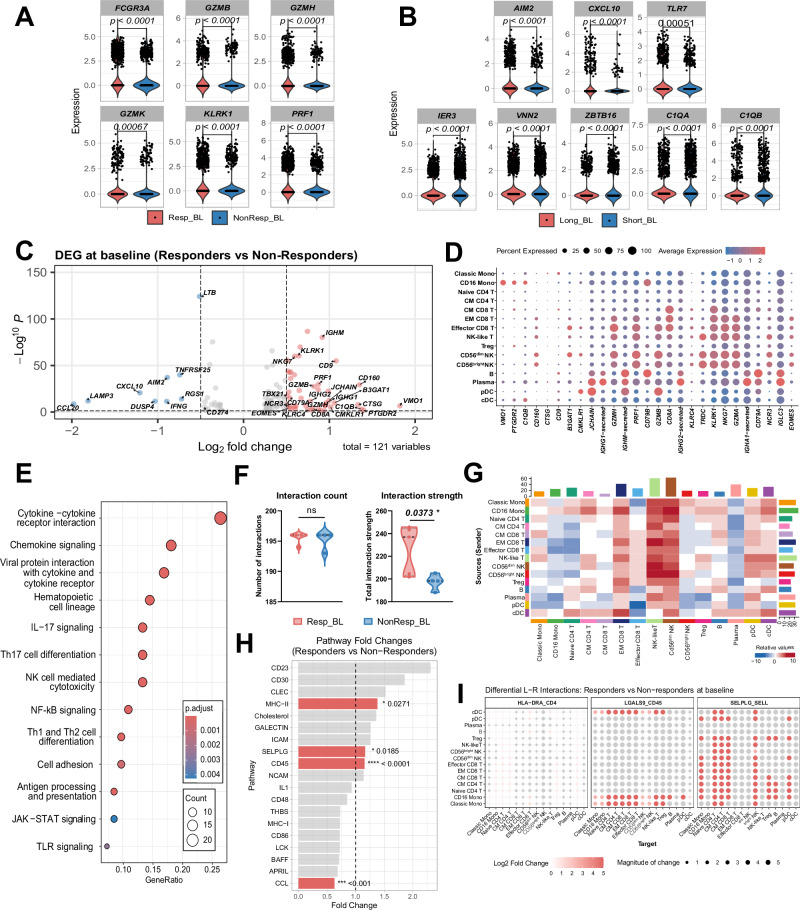
Transcriptional differences between Responders and Non-Responders at baseline (BL). **A** Transcriptional changes in Tregs comparing Responders to Non-Responders. **B** Transcriptional changes in CD16+ Monocytes comparing long survivors (OS > 12 months) to short survivors. **C** Volcano plot showing differential gene expression in PBMCs between Responders and Non-Responders. Gray dots indicate genes with *p* ≥ 0.05, with colored genes showing strongest expression differences. **D** Heatmap showing top upregulated genes per cluster in Responders at baseline. **E** KEGG pathway enrichment analysis comparing Responders to Non-Responders in PBMCs. **F** Cell-cell interaction counts and strength at baseline calculated using CellChat (v1.5.0). **G** Differential signaling patterns in Responders at baseline, with more active signaling cells shown in red and less active in blue. **H** Pathway fold changes between Responders and Non-Responders, with significant changes (*p* < 0.05) shown in red and trending differences (*p* < 0.1) in gray. **I** Ligand-receptor pair analysis showing differential interactions between cell populations, with dot size indicating interaction strength and color indicating statistical significance. Differential expression analysis was performed using Wilcoxon test. Cell-cell interaction significance was assessed using permutation tests implemented in CellChat. *p* values < 0.05 were considered significant.

Analysis of the transcriptional profiles revealed distinct differences at baseline in both Tregs and CD16+ monocyte populations, distinguishing patients who benefited from the treatment. Within the Treg cluster, we observed significantly higher expression of cytotoxicity-associated genes including *GZMB*, *GZMH*, and *GZMK* (*p* < 0.0001) in Responders compared to Non-Responders. as well as elevated expression of *KLRK1* and *PRF1* (*p* < 0.0001), suggesting an enhanced cytotoxic potential in Tregs of responding patients (Fig. 2A).

CD16+ monocytes from patients with longer survival demonstrated upregulation of pattern recognition receptor expression, including *TLR7* and *AIM2 (p* < 0.0001), suggesting enhanced capacity for detection of potential danger signals (Fig. 2B). Moreover, higher expression of *CXCL10 (p* < 0.0001) indicated an activated interferon-responsive state.

Several genes associated with tissue residency and remodeling were downregulated in longer survivors compared to short, including *C1QA*, *C1QB, VNN2 and ZBTB16 (p* < 0.0001). Downregulation of immediate early response gene *IER3*, which acts as a modulator of both inflammatory activation and cell survival pathways, suggests altered stress response regulation in these cells. This transcriptional signature indicates that circulating CD16+ monocytes in patients with longer survival maintain an enhanced danger-sensing state while showing altered tissue-homing properties that may affect their circulation time.

Overall, this data showed that several blood markers, as the abundance of Tregs or monocytes and their transcriptional profile, may reflect the baseline difference between responding and non-responding patients and potentially serve as the prognostic or predictive biomarker.

### Patients benefiting from the therapy with TILT-123 had more defined cytotoxic immune cell expression profile at the baseline

Analysis of differentially expressed genes (DEG) between Responders and Non-Responders at baseline identified 121 significant transcripts (Fig. 2C). Key upregulated genes in Responders included maturation and cytotoxic markers (*CD8A, KLRK1, KLRC4, NKG7, GZMH, GZMA, GZMB, PRF1, ZNF683, GNLY, CD160, EOMES, NCR3, TBX3*), B-cell differentiation markers (*CD79A, CD79B, MS4A1, JCHAIN, IGHM, IGHG1, IGHG2, IGHD*) and inflammation (*C1QB, VMO1, PTGDR2, CXCR1, CX3CR1, CXCR2, CXCL5, CXCL16, IL2RB*) (Fig. 2C and Supplementary Table S5). Cluster analysis confirmed that the genes with the highest fold change were mainly expressed in Effector and Effector Memory CD8 T cell, NK cells, B and plasma cells, and to lesser extent pDC and CD16 Monocytes (Fig. 2D). Further detailed analysis of gene expression in individual immune cell populations confirmed these patterns and revealed cell type-specific transcriptional differences, with particularly pronounced changes in Tregs, NK cells, and monocyte populations (Supplementary Fig. S2). A notable set of interferon-stimulated genes (ISGs) were downregulated, including *CXCL10, LAMP3, DUSP4, RGS1*, and *CD274* (PD-L1), along with additional inflammatory mediators *CCL20, IFNG, LTB*, and pro-apoptotic *TNFRSF25* (Fig. 2F). The baseline transcriptional analysis of circulating immune cells showed that Responders had higher expression of cytotoxic genes but lower type I interferon signaling and inflammatory mediators, suggesting their immune system was more favorable for response to therapy.

### Enhanced immune cell communication networks distinguish responders to TILT-123

KEGG pathway analysis revealed notable enrichment of immune cell activation in Responders compared to Non-Responders, including genes involved in signaling, cell differentiation, antigen presentation and NK cell cytotoxicity (Fig. 2E). Notably, interaction analysis revealed differences in strength but not the number of cellular interactions between Responders and Non-Responders, with Responders showing significantly stronger interaction scores (*p* = 0.0373) (Fig. 2F).

Analysis of cell-cell communication revealed that Responders exhibited significantly stronger interaction networks compared to Non-Responders at baseline, with particularly enhanced signaling involving NK cells, effector memory CD8 + T cells, and other lymphoid populations (Fig. 2G). pDC and cDC actively interacted with multiple immune cells, particularly T and B cells. Interaction analysis identified specific signaling routes significantly upregulated in Responders compared to Non-Responders, including MHC-II (*p* = 0.0271), SELPLG (*p* = 0.0185) and CD45 (*p* < 0.0001), while CCL signaling was downregulated (*p* < 0.0001) (Fig. 2H).

The underlying ligand-receptor pairs showed enhanced SELL-SELPLG, LGALS9-CD45 and HLA-DRA-CD4 interactions (Panel I). Monocytes acted as key signal senders through LGALS9, which interacted with immune cells via CD45. Memory T cells showed strong interactions with monocytes through SELL-SELPLG, important for immune cell trafficking. HLA-DR-CD4 signaling was enhanced with pDCs, CD16+ monocytes, Naïve and central memory CD4+ T cells, and Tregs as main receivers, suggesting active antigen presentation.

### Higher percentage of cytotoxic NK cells and memory T cells in peripheral blood correlated with longer survival of patients treated with TILT-123

**Fig. 3:**
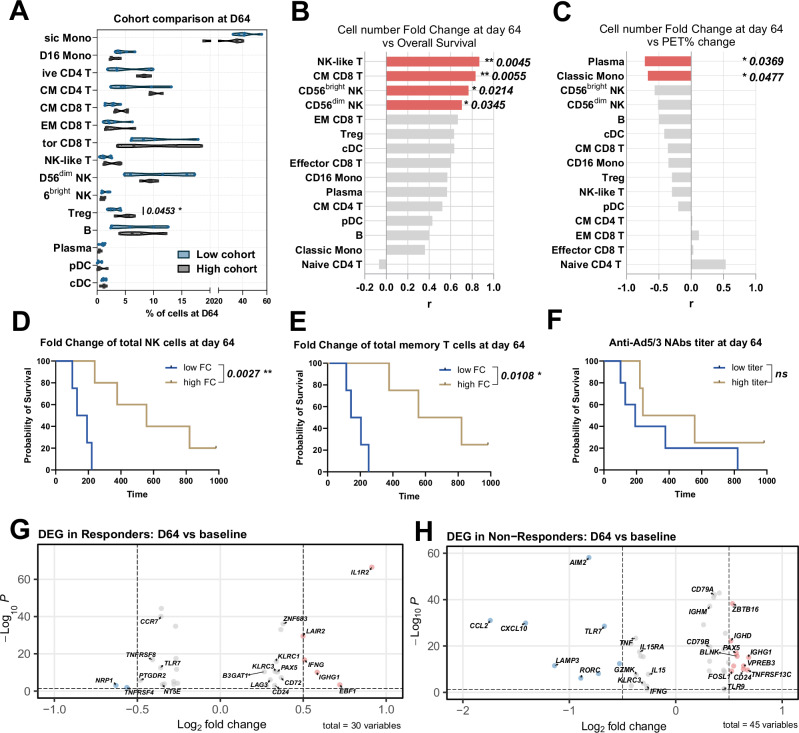
Cellular and transcriptional differences between Responders and Non-Responders after TILT-123 administration (day 64). **A** Cell type frequencies comparing patients who received lower (Cohorts 1–3, Table 1) versus higher (Cohorts 4–6, Table 1) virus doses. Data are shown as violin plots displaying distribution density with median indicated by the central line. Values are expressed as percentage of total cells per sample. **B** Correlation between cell percentage fold change at day 64 and overall survival time. **C** Correlation between cell percentage fold change at day 64 and tumor size change at day 64 (Table 1). **D** Kaplan-Meier analysis of overall survival based on NK cell fold change at day 64. **E** Kaplan-Meier analysis of overall survival based on memory T cell fold change at day 64. **F** Kaplan-Meier analysis of overall survival based on anti-Ad5/3 neutralizing antibody titers at day 64. **G** Volcano plot showing differential gene expression in Responders comparing day 64 to baseline. Gray dots indicate genes with *p* ≥ 0.05, with colored genes showing strongest expression differences. **H** Volcano plot showing differential gene expression in Non-Responders comparing day 64 to baseline. Cell frequencies were compared using unpaired *t*-test or Wilcoxon test depending on data distribution. For comparing baseline and day 64 timepoints within groups, paired *t*-test or Wilcoxon signed-rank test were used. Correlations were assessed using Pearson or Spearman correlation based on data normality, with *p* < 0.1 shown in gray and *p* < 0.05 in red. Survival analyses were performed using log-rank (Mantel–Cox) test. *p* values < 0.05 were considered significant.

Following TILT-123 therapy, blood immune cell composition at day 64 showed minor dose-dependent changes. Patients receiving higher virus doses (Cohorts 1–3; >1 × 10^11^ VP/injection) showed significantly elevated Treg percentages (*p* = 0.0453) and a trend toward increased CM CD8 T cells (*p* = 0.0637) compared to those receiving lower doses (Cohorts 4-6; <1 × 10^11^ VP/injection) (Table 1 and Fig. 3A).

However, changes in several immune cell populations at day 64 strongly correlated with clinical outcomes. Increased numbers of several lymphoid populations, including NK-like T cells (*p* = 0.0045), CM CD8 T cells (*p* = 0.0055), CD56^bright^ NK cells (*p* = 0.0214), and CD56^dim^ NK cells (*p* = 0.0345)were associated with better overall survival (Fig. 3B). Notably, changes in certain cell populations also correlated with PET response, particularly plasma cells (*p* = 0.0369) and classic monocytes (*p* = 0.0477) (Fig. 3C). The importance of NK cells and memory T cells was further validated by survival analysis, which demonstrated significantly better outcome in patients with higher fold change in these populations (Fig. 3D, E, *p* = 0.0027 and *p* = 0.0108, respectively). Interestingly, anti-Ad5/3 antibody titers at day 64 did not show significant impact on patient survival (Fig. 3F), suggesting that the development of anti-viral antibodies did not impair treatment efficacy. Overall, post-treatment analysis revealed that higher levels of cytotoxic CD56^dim^ NK cells and memory T cells correlate with longer survival and could serve as prognostic biomarkers.

### TILT-123 therapy promotes B-cell maturation and enhanced immune activation in Responders, while Non-Responders exhibit impaired immune response

Compared to baseline, fewer transcriptional changes were observed after treatment, most likely due to the intratumoral administration of the virus or collection timepoint.

Nevertheless, differential gene expression analysis of patients’ PBMCs after the TILT-123 treatment revealed 30 significantly altered genes in Responders, and 45 in Non-Responders showed (Fig. 3G, H), Responders showed upregulation of genes involved in anti-viral response *IFNG, IL1R2, KLRC1* and *KLRC3* as well as *LAIR2* and *LAG3* suggesting further stimulation and regulation of immune response on day 64. Modest yet statistically significant upregulation of B-cell markers *PAX5, EBF1, IGHG1, CD72, CD24, B3GAT1* (Fig. 3G and Supplementary Table S5) suggests B-cell maturation and activation. Additionally, downregulation of *NRP1*, *NT5E*, *TNFRSF4* and *TNFSF8* indicates a reduction in immunosuppressive stimuli in peripheral blood.

Non-Responders showed similar upregulation of B-cell markers, including *PAX5, IGHG1, IGHD, VPREB3, CD24, ZBTB16, TNFRSF13C, BLNK*, and to a lesser extend *CD79A, CD79B, TLR9* and *IGHM* (Fig. 3H and Supplementary Table S5). *GZMK*, *IL15*, *IL15RA*, *KLRC3* and *IFNG* were downregulated suggesting impaired anti-viral response. Decrease in *CXCL10*, *CCL2*, *RORC*, *LAMP3* and *AIM2* expression reveals reduced inflammatory responses in Non-Responders (Fig. 3E).

Analysis of all patients combined revealed consistent changes in immune activation pathways by day 64 (Supplementary Fig. S3). B cell-related genes showed robust upregulation, including markers of maturation (*PAX5, CD72, CD79A/B*) and immunoglobulin genes (*IGHG1, IGHM*). T cell activation was evidenced by increased expression of cytotoxicity markers (*GZMK, GZMA*), chemokine receptors (*CXCR3, CCR5*), and activation markers (*CD69, IL2RB*). Additionally, interferon-response genes showed modulation, including *STAT1, IRF4*, and interferon-stimulated chemokines. This pattern suggests TILT-123 induces broad immune system activation, though the magnitude and functional consequences of these changes differ between response groups.

Overall, our data suggests that following TILT-123 treatment, Responders demonstrated enhanced immune activation and B-cell maturation. Conversely, Non-Responders exhibited increased B-cell markers but reduced anti-viral activity, indicating potential issues with immune responses and decreased inflammation.

### Memory B cells and immunoglobulin repertoire predict response to TILT-123 and reveal treatment-induced changes

**Fig. 4:**
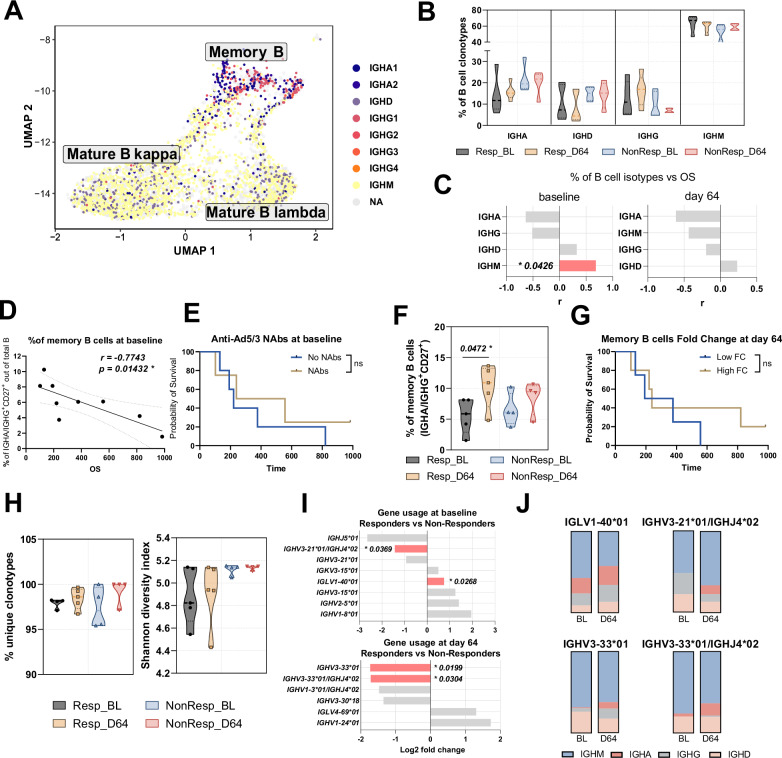
B cell receptor analysis and diversity evaluation. **A** UMAP plot showing B cell subsets based on immunoglobulin isotype expression. **B** Percentage of cells expressing different immunoglobulin isotypes (IGHA, IGHM, IGHG, IGHD) in Responders and Non-Responders at baseline and day 64. Data are shown as violin plots displaying distribution density with median indicated by the central line. Values are expressed as percentage of total B cells per sample. **C** Correlation between percentage of immunoglobulin-expressing cells and overall survival at baseline and day 64. Results with *p* < 0.1 are shown with gray and statistical significance (*p* < 0.05) is shown with red. **D** Correlation between memory B cell numbers at baseline and overall survival. **E** Kaplan-Meier analysis of overall survival based on baseline anti-Ad5/3 antibodies. **F** Memory B cell percentage at baseline and day 64. Data are shown as violin plots displaying distribution density with median indicated by the central line. Values are expressed as percentage of total B cells per sample. **G** Kaplan-Meier analysis of overall survival based on memory B cell fold change at day 64. **H** BCR clonality analysis showing percentage of unique clonotypes and Shannon diversity index in response groups at baseline and day 64. Data are shown as violin plots displaying distribution density with median indicated by the central line. Values are expressed as percentage of total B cells per sample. **I** Analysis of immunoglobulin gene segments showing differential expression between response groups. Results with *p* < 0.1 are shown with grey and statistical significance (*p* < 0.05) is shown with red. **J** Distribution of significantly differentially expressed immunoglobulin segments across cell types. Cell frequencies and clonality metrics were compared using *t*-test or Wilcoxon test depending on data distribution. For comparing baseline and day 64 timepoints within groups, paired *t*-test or Wilcoxon signed-rank test were used. Correlations were assessed using Pearson or Spearman correlation based on data normality, with *p* < 0.1 shown in grey and *p* < 0.05 in red. Survival analyses were performed using log-rank (Mantel–Cox) test. *p* values < 0.05 were considered significant.

Additional clustering analysis revealed three distinct B cell subsets: memory B cells and two mature B cell populations expressing either kappa or lambda light chains (Fig. 4A). Expression analysis of different immunoglobulin isotypes (IGHA1, IGHA2, IGHD, IGHG1-4, IGHM) showed no significant changes) (Fig. 4B). The percentage of B cell isotypes, particularly IGHA, showed changes over the course of treatment (Fig. 4C). Correlation analysis of B cell isotypes with overall survival showed that only IgM-expressing B cells at baseline had a significant positive association (Fig. 4C).

Notably, higher numbers of memory B cells at baseline negatively correlated with overall survival (*p* = 0.0453) (Fig. 4D). However, shorter survival was not due to pre-existing immunity to TILT-123, as baseline anti-Ad5/3 antibodies showed no impact on survival (Fig. 4F). Moreover, following TILT-123 treatment, both groups showed expansion of memory B cells, being statistically significant in Responders (*p* = 0.0477) but not in Non-Responders (Fig. 4F), yet this treatment-induced B cell increase did not affect patient survival (Fig. 4G).

B cell clonality analysis, based on the number of unique clonotypes and Shannon diversity index, revealed no significant differences between groups and timepoints (Fig. 4H). However, specific B cell receptor analysis identified the IGLV1-40*01 segment at baseline to be statistically positively associated with response (*p* = 0.0268), while baseline IGHV3-21*01/IGHJ4*02 joint segments as well as day 64 IGHV3-33*01 and IGHV3-33*01/IGHJ4*02 showed statistical negative association (*p* = 0.0369, *p* = 0.0199 and *p* = 0.0304, respectively) (Fig. 4I). Notably, the IGLV1-40*01 segment was predominantly found as part of class-switched antibodies (IgA, IgG), while negatively associated segments were mainly present in IgM antibodies, suggesting differences in B cell maturation state between Responders and Non-Responders (Fig. 4J). However, none of the differentially expressed segments correlated with overall survival.

### T cell receptor repertoire and pre-existing specific T cells distinguish Responders from Non-Responders

**Fig. 5:**
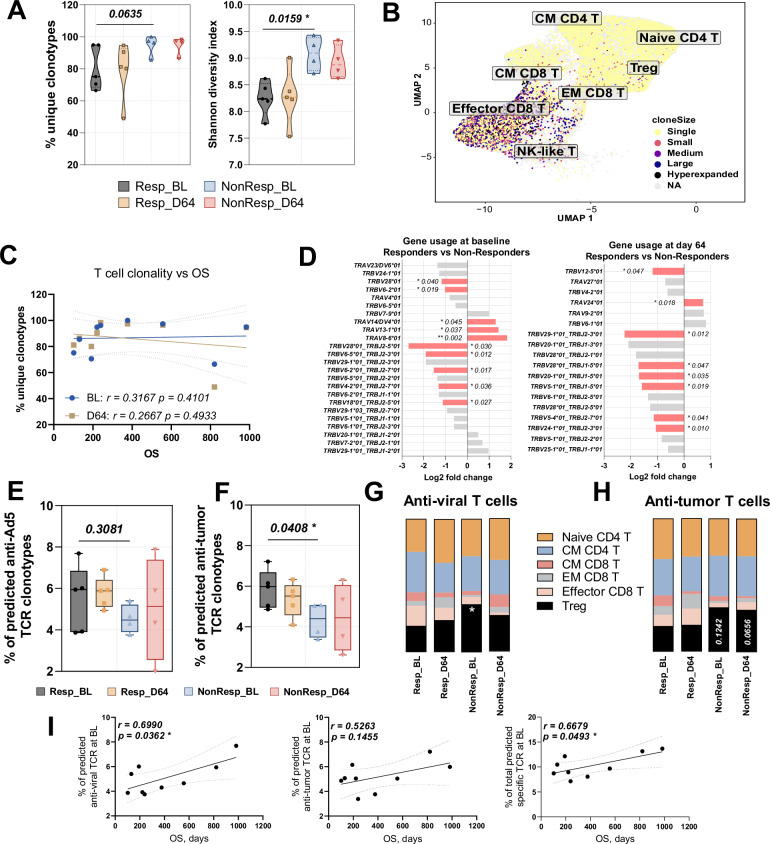
T cell receptor analysis and predicted antigen specificity. **A** TCR clonality analysis showing percentage of unique clonotypes and Shannon diversity index in response groups at baseline and day 64. Data are shown as violin plots displaying distribution density with median indicated by the central line. Values are expressed as percentage of total T cells per sample. **B** UMAP plot showing distribution of TCR clones across T cell subsets. Clone size is defined as follows: Single—*X* = 1, Small—1 < *X* ≤ 5, Medium—5 < *X* ≤ 10, Large—10 < *X* ≤ 50, Hyperexpanded—*X* > 50. **C** T cell clonality correlation with overall survival at baseline and day 64. **D** Analysis of TCR segments showing differential expression between response groups. **E** Percentage of predicted anti-Ad5 specific T cells in Responders and Non-Responders at baseline and day 64. **F** Percentage of predicted anti-tumor specific T cells in Responders and Non-Responders at baseline and day 64. **G** Distribution of predicted anti-Ad5 TCRs across T cell subsets. **H** Distribution of predicted anti-tumor TCRs across T cell subsets. **I** Correlation between percentage of specific T cells at baseline and overall survival. Cell frequencies and clonality metrics were compared using *t*-test or Wilcoxon test depending on data distribution. For comparing baseline and day 64 timepoints within groups, paired *t*-test or Wilcoxon signed-rank test were used. Correlations were assessed using Pearson or Spearman correlation based on data normality. *p* values < 0.05 were considered significant.

Analysis of T cell receptor diversity indicates that Responders showed a trend towards a lower percentage of unique clonotypes at both time points (*p* = 0.0635) while, more precise Shannon diversity index showed significantly lower diversity at baseline (*p* = 0.0159), indicating presence of expanded clones (Fig. 5A). UMAP visualization showed that these expanded clones were mainly found in Effector CD8 T cells, while memory T cells and Tregs mostly contained single clones (Fig. 5B). However, this pattern of T cell clone distribution showed no correlation with overall survival at either baseline or day 64 (Fig. 5C).

Comparison of TCR sequences between Responders and Non-Responders identified distinct patterns at different timepoints. At baseline, 11 segments showed significant negative association with response (*p* < 0.05), with TRBV28*01/TRBJ2-5*01, TRBV6-5*01/TRBJ2-3*01, and TRBV13-1*01 showing the strongest differences, while TRAV8-6*01 was strongest positively associated with response (*p* = 0.002) (Fig. 5D, left panel). At day 64, 10 segments were differentially expressed, with TRBV29-1*01/TRBJ2-3*01 showing the largest fold change (*p* = 0.012). Notably, TRBV28*01/TRBJ2-5*01 maintained its negative association across both timepoints and TRBV28*01 appeared in multiple negatively associated joint segments at day 64 (Fig. 5D, right panel). The predominant negative association of TCR segments with response likely reflects focused clonal expansion in Responders, where specific expanded clones dominate the repertoire while other TCR segments show relatively lower expression.

TCR specificity was evaluated via prediction of their binding to known adenoviral and selected cancer epitopes. Each patient had several predicted anti-Ad5 TCRs both before and after the treatment, with a trending increase in their numbers following TILT-123 administration (Fig. 5E). Interestingly, Responders tended to have a higher number of predicted adenovirus-specific T cells at baseline. Moreover, analyzing the matching adenoviral peptides we found that the majority of TCRs were highly specific to two adenoviral peptides: NWAAFRGWAF from hexon protein (45.8% of matches) and NRILLEQAAITTTPR from pre-hexon-linking protein VIII (37.5% of matches) (Supplementary Table S6).

Analysis of predicted antitumor TCRs showed significantly higher baseline percentage in Responders compared to Non-Responders (*p* = 0.0408) (Fig. 5F). Predicted matching epitopes included various cancer antigens, including FOXM1 (IYTWIEDHF), mucin 1 (LLLLTVLTV), Cancer/testis antigen 1 (AMPFATPMEA, GAARASGPGGGAPRG) and ADAM10 (GQYGNPLNK) (Supplementary Table S7). Cell type analysis showed that antiviral and antitumor TCRs were primarily expressed on Central Memory CD4 T cells and Tregs (Fig. 5G, H). Additionally, Responders showed higher numbers of specific Effector and Effector Memory CD8 T cells at both timepoints compared to Non-Responders. The percentage of predicted antiviral Tregs in Non-Responders was significantly higher at baseline (*p* = 0.0271) compared to Responders (Fig. 5G), while the proportion of predicted antitumor TCRs showed higher trend in Responders both before (*p* = 0.1242) and after (*p* = 0.0656) the treatment (Fig. 5H). Correlation analysis revealed significant positive association between overall survival and percentage of predicted antiviral T cells (*p* = 0.0362) as well as combined antiviral and antitumor T cells (*p* = 0.0493), while antitumor TCRs showed a positive trend (*p* = 0.1455) (Fig. 5I).

Overall, TCR analysis showed that Responders had more focused TCR repertoire with specific expanded clones, while Non-Responders showed higher proportion of anti-viral and anti-tumor Tregs, suggesting that pre-existing immunosuppressive environment might affect treatment outcome.

## Discussion

Oncolytic viruses have emerged as a promising strategy in cancer therapy. Recent years have seen a significant increase in the number of clinical trials investigating OVs across various types of solid cancers, reflecting mounting interest and investment in this area of research [13, 14, 27, 28]. Moreover, a growing number of trials suggests that OVs are versatile therapies that can be combined with other therapeutic modalities, such as immune checkpoint inhibitors and adoptive-cell therapies, to enhance antitumor effects [29, 30].

The efficacy of oncolytic virus therapy is not uniform across all patients, and emerging evidence indicates that the baseline immune status of a patient plays a critical role in determining their response to the treatment [14, 31]. Notably, the presence of a robust immune system at baseline has been recently associated with superior responses to oncolytic viruses in preclinical and clinical settings [32, 33].

Our research contributes to the expanding field of cancer therapies by analyzing immune changes in the peripheral blood at the single-cell level in patients treated with the oncolytic adenovirus TILT-123 coding for IL2 and TNFα. This analysis provides deep insights into the variability of responses seen among patients and the possibilities for future therapeutic improvements.

Analysis of baseline immune profiles revealed distinctly different immune states between Responders and Non-Responders. Already at baseline, Responders showed higher expression of cytotoxic markers, particularly granzymes and perforin, and enhanced inflammatory signaling. This active immune state was further evidenced by significantly stronger cell-cell communication networks, particularly involving NK cells, effector memory CD8+ T cells, and dendritic cells. Notably, Responders demonstrated downregulation of type I interferon response pathway as well interferon gamma suggesting a less restrictive antiviral response that could allow for more effective TILT-123 propagation upon administration [34].

Our peripheral blood analysis complements previous single-cell studies that focused primarily on tumor samples in oncolytic virus trials. While Ramelyte et al. and Zhang et al. demonstrated rapid activation of NK cells and dendritic cells in tumors following T-VEC and H101 treatment respectively [13, 14], our findings suggest that systemic immune signatures, particularly enhanced baseline cell-cell communication networks and cytotoxic potential, may predict treatment outcomes. These patterns parallel observations from checkpoint blockade studies, where pre-existing immune activation emerged as a key response determinant [35, 36]. Several specific cell populations emerged as particularly relevant for treatment outcomes. CD16 monocytes showed notable correlation with survival, with higher baseline levels in patients achieving longer survival. These cells demonstrated enhanced expression of pattern recognition receptors and reduced tissue-homing properties, potentially extending their circulation time and anti-tumor activity through mechanisms such as antibody-dependent cellular cytotoxicity [37]. The observed decrease in CD16+ monocytes in peripheral blood over time, particularly in responding patients, could indicate trafficking of these cells to tumor sites, though direct evidence would require matched tumor sample analysis. Their precise contribution—whether primarily antiviral or antitumor—remains to be elucidated. Future mechanistic studies using targeted blocking experiments could help distinguish these functions and further refine patient stratification strategies.

In contrast, Non-Responders showed higher baseline levels of Tregs, known for their immunosuppressive functions that can inhibit effective antitumor responses [38]. Moreover, recent studies showed that the higher presence of Tregs in blood before the immunotherapy treatment predicted tumor recurrency [39]. Notably, the expression profile of Tregs in Responders resembled that of inflammatory exTreg cells which possess rather cytotoxic phenotype [40]. Furthermore, the expansion of specific antiviral—and to a lesser extent, antitumor—Tregs observed in Non-Responders at baseline suggests that this group of patients may experience limited benefits from the treatment.

Further characterization of T cell populations focused on their receptor diversity, where showing that responding patients had lower diversity of TCRs at baseline, suggesting their clonal expansion. It is still debatable whether higher clonality is beneficial for the therapeutic outcome, and whether there is any association with the cancer type. Moreover, the origin of the expanded T cells (peripheral or intratumoral) could significantly impact the outcome prediction [41]. While numerous studies reported that higher clonality of circulating T cells at baseline may contribute to the improved survival in patients receiving various types of immunotherapies and may serve as a non-invasive predictive marker of response in certain types of cancer [35, 36, 42], our study showed no correlation with survival, suggesting that TCR clonality might not serve as a universal response predictor. Specific TCR segment analysis revealed several differentially expressed segments between Responders and Non-Responders, with TRBV28*01-containing segments maintaining negative association across both timepoints. Interestingly, this segment was previously reported to be associated with the response to SARS-CoV-2 infection [43], suggesting possible cross-reactivity. However, it should be noted that TCR segment analysis has advanced significantly during the SARS-CoV-2 pandemic, and data about other viral or pathogen-specific segments remains limited. More comprehensive analysis of T cell specificity would be needed to understand these associations in context of oncovirotherapy.

To determine the specificity of the captured TCRs, we searched publicly available databases like VDJdb [44] or CEDAR [24], but no exact matches were previously published. However, using prediction tools we observed a trending increase in baseline proportion of T cells with predicted anti-adenoviral specificity and statistically higher number of T cells with predicted anti-tumor specificity in Responders suggesting their more focused antigen-specific immunity.

Immunoglobulin profiling showed similar isotype distribution between response groups. However, we observed that baseline levels of class-switched memory B cells were elevated in patients with poorer survival outcomes. Importantly, this negative prognostic value was not linked to anti-adenoviral immunity, as pre-treatment anti-Ad5/3 antibodies showed no impact on survival.

Regarding BCR repertoire, while it has been shown that in cancer BCR diversity can be associated with mutation load, tumor stage, or age and serve as a survival prognostic factor [45], our B cell receptor clonality analysis showed no differences between response groups. However, examination of immunoglobulin gene segments revealed differential expression of specific combinations, particularly IGLV1-40*01 showing positive association with response while three *IGHV3*-related segments were negatively associated. Notably, similarly to TCR data, these *IGHV3* segments were previously identified as components of neutralizing antibodies in patients with severe SARS-CoV-2 infection and might be involved in the humoral immune responses against it [46]. This suggests an opportunity for future research to explore whether the frequency of certain segments and/or genes correlates with antiviral response towards specific viruses, or whether baseline immunity towards other infections may contribute to the efficacy of OVs therapy.

Overall, while previous studies indicated that pre-existing immunity to oncolytic viruses might enhance therapeutic efficacy [32, 47], our findings suggest a more nuanced relationship between different components of the immune system. The type of pre-existing immune response appears crucial: while higher numbers of virus-specific T cells at baseline associated with better outcomes, elevated memory B cells correlated with poorer survival. Moreover, the presence of TCR and BCR segments previously linked to SARS-CoV-2 responses suggests that cross-reactivity might play an important role. These observations emphasize the need to carefully evaluate not only the presence but also the nature of pre-existing immunity, distinguishing between cellular and humoral responses. This complexity extends to innate immune populations as well. For instance, while CD16+ monocytes showed strong association with treatment outcomes, their precise contribution—whether primarily antiviral or antitumor—remains to be elucidated, similar to the dual role observed with T cells. Future mechanistic studies could help distinguish these functions across different immune cell populations and refine patient stratification strategies.

Following the assessment of the immune status, we analyzed how changes in immune cell populations correlated with clinical outcomes. Notably, higher fold change in NK cells and memory T cells at day 64 significantly associated with better overall survival. Similar positive correlation was observed with changes in cytotoxic NK cells, central memory CD8+ and CD4+ T cells. As key players in innate immunity, NK cells are rapidly activated by viral infections and recruited to affected tissues, providing immediate immune response [48]. This innate response is then supported by development of adaptive immunity, where memory T cells ensure long-term protection. Additionally, changes in plasma cells correlated with reduced tumor size according to PET evaluation, while their increased activity was reflected in upregulation of B cell maturation markers and immunoglobulin-related genes in both groups. Interestingly, despite the active B cell response to viral treatment, anti-Ad5/3 antibody levels at day 64 did not affect patient survival.

However, while we observed these specific population changes, the overall magnitude of peripheral immune alterations was relatively modest, likely reflecting both the predominantly local nature of intratumoral administration and potential temporal limitations of our day 64 sampling timepoint. The more pronounced baseline differences between responders and non-responders suggest that pre-existing systemic immunity may be more predictive of outcomes than treatment-induced peripheral changes.

Overall, in this study we identified important signatures that distinguished patients responding to TILT-123 therapy from non-responders, providing important insights into treatment efficacy. While more homogeneous patient cohorts could potentially reveal tumor type-specific response patterns with greater statistical power, analysis of heterogeneous patient populations has proven valuable in early-phase oncolytic virus trials, as demonstrated by Garcia-Carbonero et al. studying VCN-01 treatment across different solid tumors [47]. This broad approach allows identification of universal response mechanisms and helps define patient populations for subsequent focused studies. Moving forward, addition of intratumoral samples could enhance our understanding of immune cell trafficking and local responses, complementing our peripheral blood analysis. Furthermore, analysis of more homogeneous patient cohorts would help reveal cancer type-specific patterns, while expanding the number of patients and sampling timepoints would allow better characterization of immune response dynamics.

## Supplementary information

Supplemental Figure S1

Supplemental Figure S2

Supplemental Figure S3

Supplemental Table S1

Supplemental Table S2

Supplemental Table S4

Supplemental Table S6

Supplemental Table S7

Supplemental Table S3

Supplemental Table S5

## Data Availability

All data are available in the main text or Supplementary materials. Raw patients’ data will be shared and distributed for research purposes upon reasonable request to the corresponding author. The processed expression matrices can be accessed in the ArrayExpress database under the accession number E-MTAB-14799.
