# Supplementary material for: Single-cell profiling of peripheral blood mononuclear cells from patients treated with oncolytic adenovirus TILT-123 reveals baseline immune status as a predictor of therapy outcomes
Source: Cancer Gene Ther. 2025 Apr 10;32(6):649–61. doi: 10.1038/s41417-025-00901-z (PMC12183079; doi:10.1038/s41417-025-00901-z)
Supplement: Supplementary file 1 — Supplemental Figure S1 [file 41417_2025_901_MOESM1_ESM.pdf]

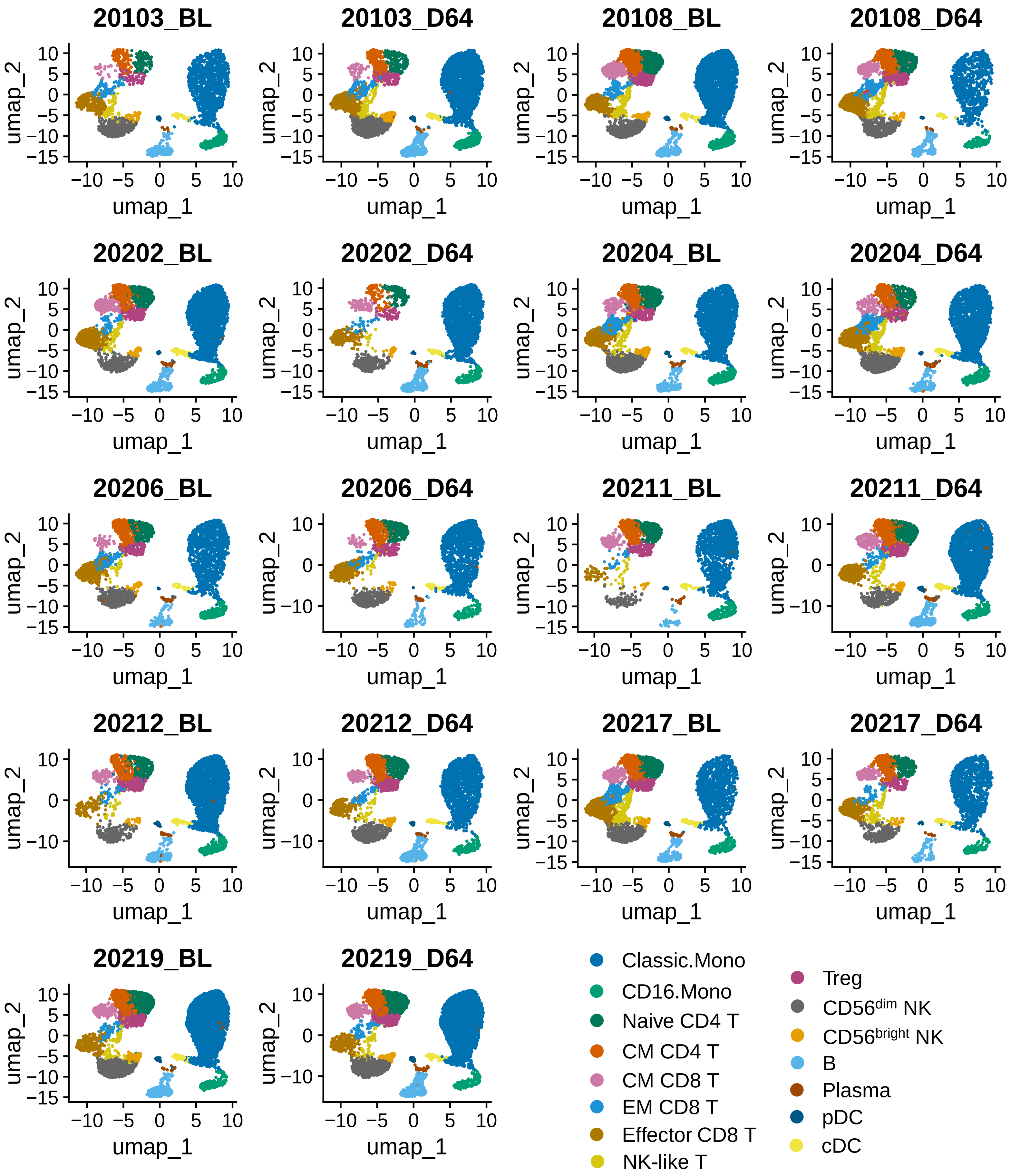

Supplemental Figure S1. Individual UMAP plots of baseline and day 64 PBMCs collected from patients before (BL) and after (D64) they were administered with TILT-123. CM CD4 T = Central Memory CD4 T cells, CM CD8 T = Central Memory CD8 T cells, EM CD8 T = Effector Memory CD8 T cells, Treg = T regulatory cells, cDC = conventional dendritic cells, pDC = plasmacytoid dendritic cells.
