# Supplementary material for: Single-cell profiling of peripheral blood mononuclear cells from patients treated with oncolytic adenovirus TILT-123 reveals baseline immune status as a predictor of therapy outcomes
Source: Cancer Gene Ther. 2025 Apr 10;32(6):649–61. doi: 10.1038/s41417-025-00901-z (PMC12183079; doi:10.1038/s41417-025-00901-z)
Supplement: Supplementary file 2 — Supplemental Figure S2 [file 41417_2025_901_MOESM2_ESM.pdf]

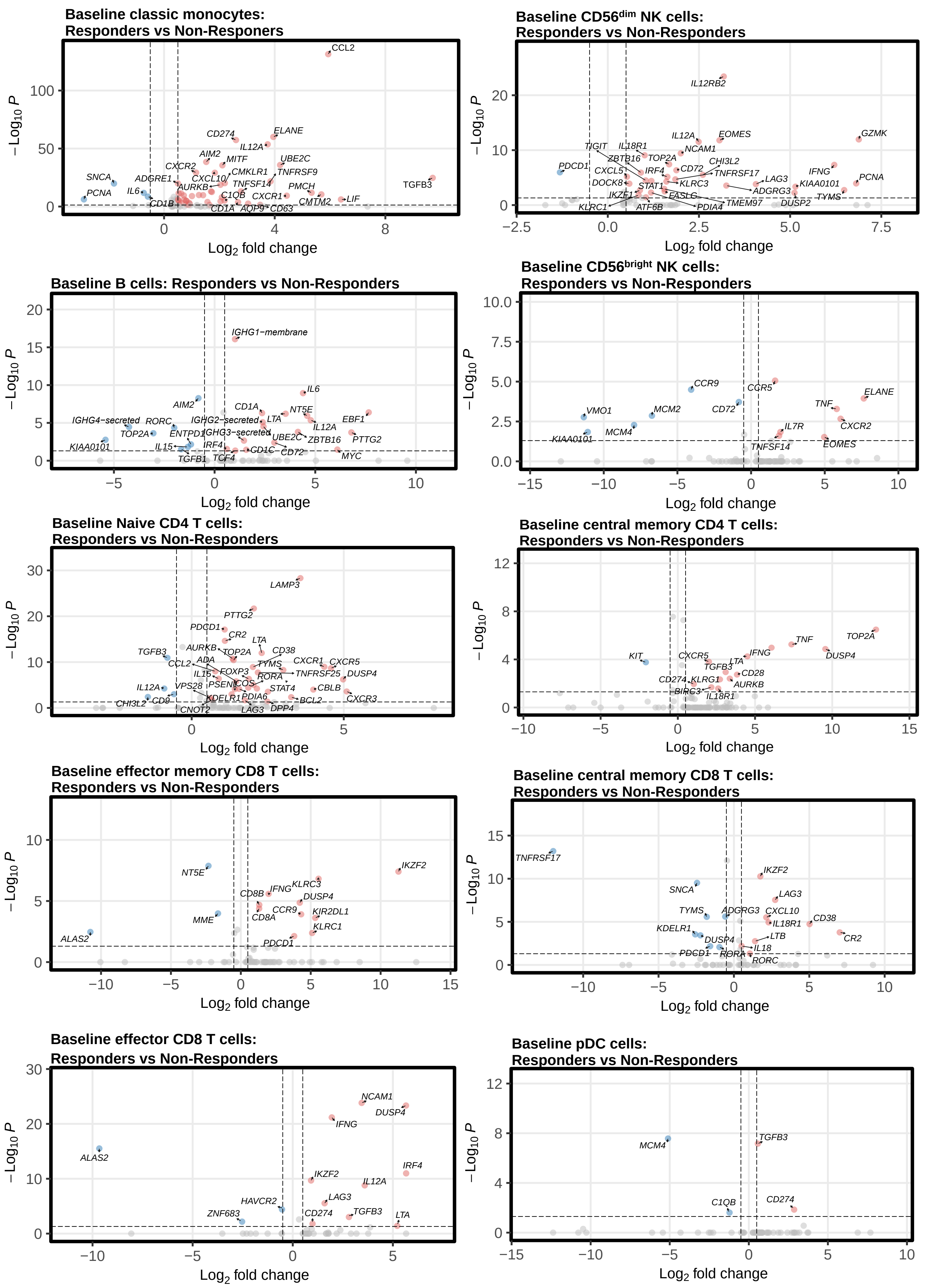

Supplemental Figure S2.

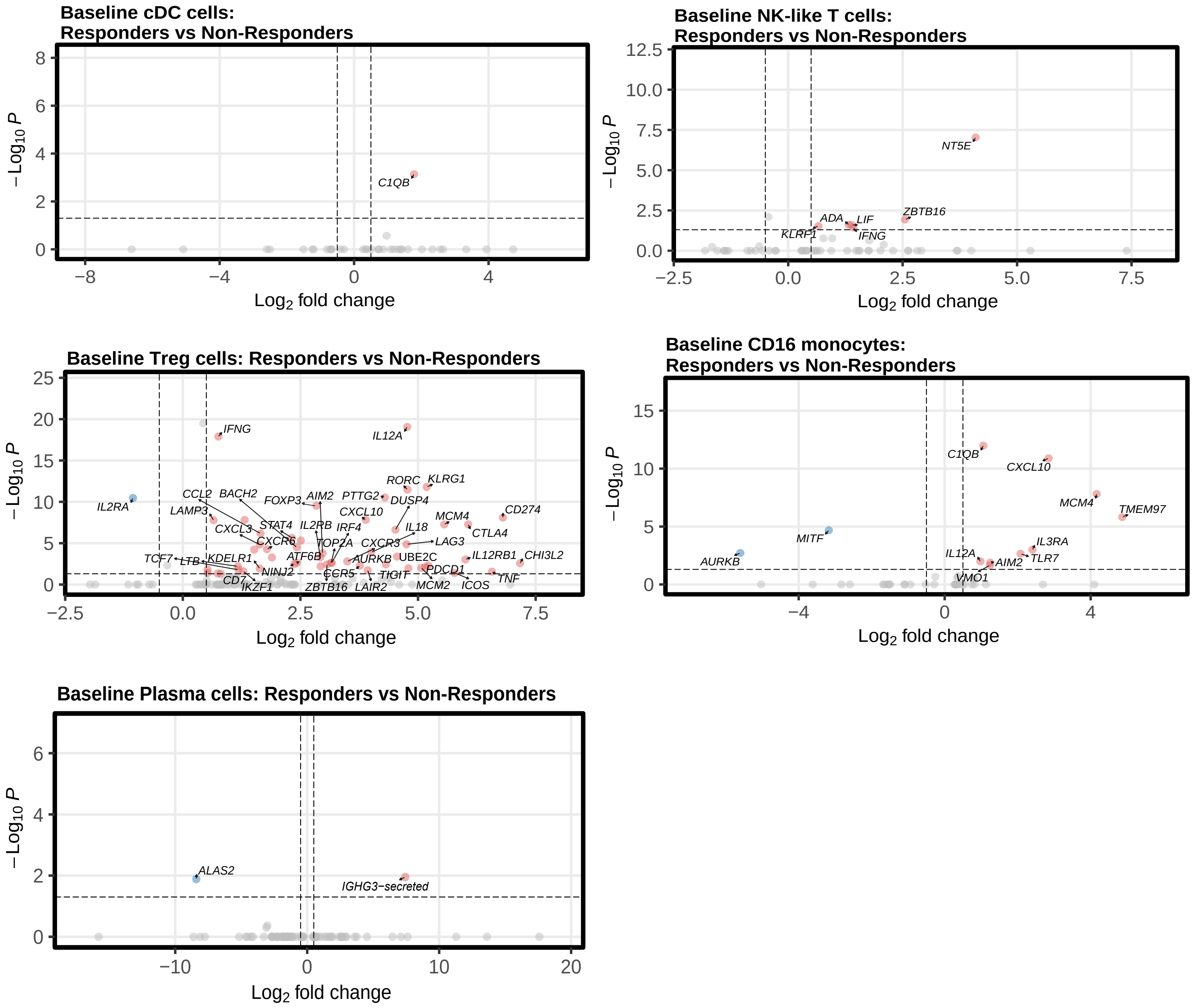

Supplemental Figure S2. Volcano plot showing differential gene expression between Responders and Non-Responders in each cluster of immune cells individually. Grey dots indicate genes with p≥0.05, with colored genes showing strongest expression differences.
