# Supplementary material for: Single-cell profiling of peripheral blood mononuclear cells from patients treated with oncolytic adenovirus TILT-123 reveals baseline immune status as a predictor of therapy outcomes
Source: Cancer Gene Ther. 2025 Apr 10;32(6):649–61. doi: 10.1038/s41417-025-00901-z (PMC12183079; doi:10.1038/s41417-025-00901-z)
Supplement: Supplementary file 3 — Supplemental Figure S3 [file 41417_2025_901_MOESM3_ESM.pdf]

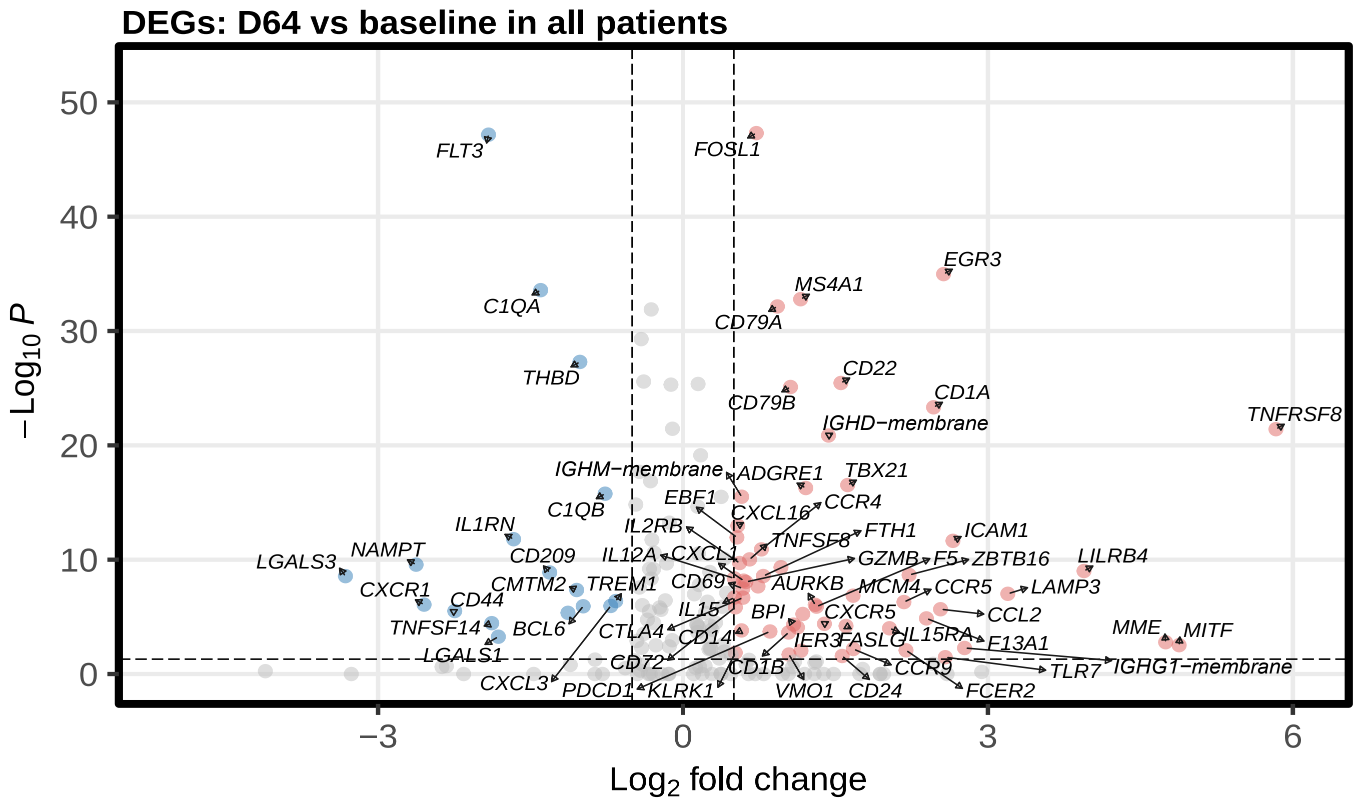

Supplemental Figure S3. Volcano plot showing differential gene expression in PBMCs comparing day 64 after TILT-123 treatment initiation and baseline in all patients. Grey dots indicate genes with p≥0.05, with colored genes showing strongest expression differences.
