# Supplementary material for: Single-cell profiling of peripheral blood mononuclear cells from patients treated with oncolytic adenovirus TILT-123 reveals baseline immune status as a predictor of therapy outcomes
Source: Cancer Gene Ther. 2025 Apr 10;32(6):649–61. doi: 10.1038/s41417-025-00901-z (PMC12183079; doi:10.1038/s41417-025-00901-z)
Supplement: Supplementary file 4 — Supplemental Table S1 [file 41417_2025_901_MOESM4_ESM.pdf]

| Response rate | Description |
|---|---|
| Complete metabolic response – CMR | Complete resolution of FDG activity within measurable lesions and all reliably assessable lesions to background levels. No new FDG-avid lesions in pattern typical of cancer*. Lymph nodes may remain metabolically active due to immune response (activated lymphocytes take up FDG). |
| Partial metabolic response – PMR | > 30 percent decrease in FDG activity measured as the summed SUVmax of measurable lesions (up to five lesions, max 2/organ). No new FDG-avid lesions in pattern typical of cancer*. |
| Minor metabolic response – MMR | 10-29 percent decrease in FDG summed SUVmax. No new FDG-avid lesions in pattern typical of cancer*. |
| Stable metabolic disease – SMD | 0-9 percent decrease or up to < 30 percent increase in FDG summed SUVmax. No new FDG-avid lesions in pattern typical of cancer*. |
| Progressive metabolic disease – PMD | ≥ 30 percent increase in FDG summed SUVmax in pattern typical of tumor, or new clearly FDG-avid clinically significant lesions in pattern typical of cancer*. Increase in metabolic activity in lymph nodes should not result in PMD if no progression is detected elsewhere, since it might reflect immunological activation and not progression. |
| * "clinically significant lesions in pattern typical of cancer" defined as lesions associated with a CT abnormality most consistent with cancer (≥ 2 cm in diameter), and clearly not because of inflammation of infection or related to treatment response. | |

**Supplemental Table S1.** PET criteria used in TUNIMO.
