# Supplementary material for: Single-cell profiling of peripheral blood mononuclear cells from patients treated with oncolytic adenovirus TILT-123 reveals baseline immune status as a predictor of therapy outcomes
Source: Cancer Gene Ther. 2025 Apr 10;32(6):649–61. doi: 10.1038/s41417-025-00901-z (PMC12183079; doi:10.1038/s41417-025-00901-z)
Supplement: Supplementary file 5 — Supplemental Table S2 [file 41417_2025_901_MOESM5_ESM.pdf]

	Batch 1 (n=6)						Batch 2 (n=6)						Batch 3 (n=6)					
ID	20202_BL	20202_D64	20204_BL	20204_D64	20206_BL	20206_D64	20103_BL	20103_D64	20108_BL	20108_D64	20212_BL	20212_D64	20211_BL	20211_D64	20217_BL	20217_D64	20219_BL	20219_D64
Cell number to load on cartridge	10000	10000	10000	10000	10000	10000	10000	10000	10000	10000	10000	10000	10000	10000	10000	10000	10000	10000
Cell number recovered from cartridge	41065						41159						41540					
**mRNA**																		
Aligned Reads	703,780,592						811,682,365						747,542,327					
Putative Cell Count	29,973						30,832						33,718					
Mean Reads per Cell	17,667.98						24,012.83						20,431.05					
Mean Molecules per Cell	1,353.49						804.96						752.74					
Mean Bioproducts per Cell	147.41						113.58						107.71					
Total Bioproducts Detected	396						395						394					
Sequencing Saturation (%)	95.26%						99.03%						99,13%					
**Sample Tag**																		
Aligned Reads	4,729,421	2,812,936	3,252,184	5,314,278	2,674,654	4,203,675	2,139,660	8,799,774	6,446,671	3,293,962	1,964,908	5,394,797	3,938,142	7,333,602	3,761,158	1,732,338	4,906,276	5,076,533
Putative Cell Count	3,137	4,134	2,864	3,369	4,983	4,781	2,929	5,632	5,363	3,820	3,962	3,658	2,735	5,086	5,361	2,916	5,698	5,724
Mean Reads per Cell	667.99	290.88	244.06	427.07	322.47	568.82	400.44	889.74	603.61	334	264.18	792.58	608.67	636.17	334.95	307.12	494.83	478.46
**VDJ TCR**																		
Aligned Reads	11,004,844						76,375,458						74,866,493					
Mean Reads per Cell	169.27						1,670.91						1,479.21					
Mean Molecules per Cell	4.36						1.02						0.83					
Moleculer VDJ Annotated	602,716						118,125						77,153					
**VDJ BCR**																		
Aligned Reads	2,525,787						36,485,468						34,824,218					
Mean Reads per Cell	50.84						851.34						801.43					
Mean Molecules per Cell	5.98						1.83						1.78					
Moleculer VDJ Annotated	278,567						114,164						116,414					

Supplemental Table S2. Sequencing outcome characteristics
