# Supplementary material for: Single-cell profiling of peripheral blood mononuclear cells from patients treated with oncolytic adenovirus TILT-123 reveals baseline immune status as a predictor of therapy outcomes
Source: Cancer Gene Ther. 2025 Apr 10;32(6):649–61. doi: 10.1038/s41417-025-00901-z (PMC12183079; doi:10.1038/s41417-025-00901-z)
Supplement: Supplementary file 6 — Supplemental Table S4 [file 41417_2025_901_MOESM6_ESM.pdf]

Sample name	Classic Mono	CD16 Mono	Naïve CD4 T	CM CD4 T	CM CD8 T	EM CD8 T	Effector CD8 T	$CD56^{dim}$ NK	$CD56^{bright}$ NK	NK-like T	Treg	B	Plasma	pDC	cDC
**20103_BL**	40.64	8.81	3.09	3.53	0.82	4.05	9.19	14.27	1.41	2.81	2.33	6.65	0.21	0.72	1.47
**20103_D64**	36.91	6.71	1.82	4.83	1.37	3.16	8.24	17.29	2.27	2.38	2.36	9.98	0.30	0.83	1.58
**20108_BL**	37.44	4.47	8.35	10.49	7.52	1.80	5.97	9.66	1.03	3.10	3.87	3.83	0.41	0.43	1.63
**20108_D64**	18.88	2.49	8.55	11.53	5.37	6.66	18.46	9.20	0.73	4.25	6.58	6.24	0.34	0.13	0.58
**20202_BL**	44.61	4.38	7.33	6.63	5.15	1.79	10.01	5.66	0.85	1.50	3.53	5.95	0.73	0.27	1.62
**20202_D64**	55.82	4.11	3.51	2.39	2.93	1.08	6.25	5.90	0.80	0.41	1.79	12.59	1.05	0.19	1.18
**20204_BL**	36.75	7.55	4.64	5.53	2.53	4.96	10.52	12.70	2.34	1.99	3.68	4.46	0.61	0.40	1.36
**20204_D64**	32.13	5.22	3.45	4.26	2.71	6.26	17.88	15.91	1.81	2.61	2.97	2.61	0.80	0.26	1.12
**20206_BL**	30.82	6.29	8.52	10.42	1.48	3.68	14.55	13.81	0.98	0.95	4.07	2.55	0.98	0.09	0.80
**20206_D64**	41.62	3.70	9.22	9.63	1.40	2.16	10.20	11.63	0.98	0.98	4.16	2.44	1.05	0.03	0.80
**20211_BL**	42.32	7.66	13.14	13.29	5.95	1.36	1.94	3.85	0.43	0.89	4.90	1.28	0.70	0.93	1.36
**20211_D64**	41.18	3.16	9.93	13.15	4.18	1.92	5.96	4.84	0.85	1.20	4.10	5.79	1.37	0.83	1.55
**20212_BL**	56.20	4.69	6.85	5.75	2.39	1.39	2.97	4.44	0.78	0.73	5.62	5.37	0.55	0.40	1.86
**20212_D64**	35.54	3.42	8.11	9.36	3.34	1.79	6.54	9.63	1.35	1.16	5.57	12.20	0.41	0.33	1.24
**20217_BL**	20.52	2.62	6.51	9.88	4.36	5.76	18.78	11.78	0.89	6.51	5.11	4.04	1.07	0.43	1.74
**20217_D64**	35.16	4.08	7.14	9.41	5.36	3.13	13.96	7.74	0.67	3.73	3.16	3.98	0.39	0.50	1.60
**20219_BL**	43.86	2.33	8.83	10.80	3.27	1.67	3.85	9.05	1.58	1.49	5.78	4.87	0.65	0.80	1.16
**20219_D64**	40.14	2.34	9.46	9.98	3.11	1.47	3.65	10.73	1.16	1.40	5.01	7.92	0.68	1.77	1.18
**Overall**															
**Resp_BL**	38.05	6.30	6.39	7.32	3.50	3.26	10.05	11.22	1.32	2.07	3.50	4.69	0.59	0.38	1.38
**Resp_D64**	37.07	4.45	5.31	6.53	2.76	3.87	12.20	11.99	1.32	2.13	3.57	6.77	0.71	0.29	1.05
**NonResp_BL**	40.72	4.32	8.83	9.93	3.99	2.54	6.89	7.28	0.92	2.41	5.35	3.89	0.74	0.64	1.53
**NonResp_D64**	38.00	3.25	8.66	10.47	4.00	2.08	7.52	8.24	1.01	1.87	4.46	7.47	0.71	0.86	1.39

Supplemental Table S4. Cell percentage for each cluster of immune cells captured from patients PBMCs. CM CD4 T = Central Memory CD4 T cells, CM CD8 T = Central Memory CD8 T cells, EM CD8 T = Effector Memory CD8 T cells, Treg = T regulatory cells, cDC = conventional dendritic cells, pDC = plasmacytoid dendritic cells, BL = baseline, D64 = day 64 after treatment initiation, Resp = Responders, NonResp = Non-Responders.
