# Supplementary material for: Single-cell profiling of peripheral blood mononuclear cells from patients treated with oncolytic adenovirus TILT-123 reveals baseline immune status as a predictor of therapy outcomes
Source: Cancer Gene Ther. 2025 Apr 10;32(6):649–61. doi: 10.1038/s41417-025-00901-z (PMC12183079; doi:10.1038/s41417-025-00901-z)
Supplement: Supplementary file 7 — Supplemental Table S6 [file 41417_2025_901_MOESM7_ESM.pdf]

IEDB ID	Peptide	Gene	Count	%
98310	NWAAFRGWAF	Hexon protein	184	46
45685	NRILLEQAAITTTPR	Pre-hexon-linking protein VIII	152	38
98592	VPFHIQVPQKFFAIKNLLLLPGSYT	Hexon protein	22	5.5
134331	AYPANFPYPLIGKTA	Hexon protein	21	5.25
69394	VLAWTRAFV	DNA polymerase	7	1.75
54790	RLTLRFIPV	Hexon protein	3	0.75
134318	AFRGWAFTRLKTKET	Hexon protein	2	0.5
98625	YDYMNKRVVA	Hexon protein	2	0.5
134354	EDTAYSYKARFTLAV	Hexon protein	1	0.25
22612	GTAYNALAPKGAPNP	Hexon protein	1	0.25
26655	IIRFDENGVLLNNSF	Fiber protein	1	0.25
98120	IPYLDGTFY	Hexon protein	1	0.25
45143	NNKFRNPTVAPTHDV	Hexon protein	1	0.25
52862	QWSYMHISGQDASEY	Hexon protein	1	0.25
98634	YKDYQQVGILHQHNNSGFVGYLAPT	Hexon protein	1	0.25

Supplemental Table S6. Adenovirus 5 peptides predicted to bind to T cell receptors detected in the study and the number of TCR-peptide matches
