# Supplementary material for: Single-cell profiling of peripheral blood mononuclear cells from patients treated with oncolytic adenovirus TILT-123 reveals baseline immune status as a predictor of therapy outcomes
Source: Cancer Gene Ther. 2025 Apr 10;32(6):649–61. doi: 10.1038/s41417-025-00901-z (PMC12183079; doi:10.1038/s41417-025-00901-z)
Supplement: Supplementary file 8 — Supplemental Table S7 [file 41417_2025_901_MOESM8_ESM.pdf]

IEDB ID	Peptide	Gene	Count	%
46714	IYTWIEDHF	Forkhead box protein M1	65	17.96
1853231	IFQEMFDKHYK	neoantigen: Isocitrate dehydrogenase [NADP], mitochondrial	63	17.40
223378	LRVAPEEHPVL	Actin, cytoplasmic 1	48	13.26
181328	GQYGNPLNK	Disintegrin and metalloproteinase domain-containing protein 10 (ADAM10)	37	10.22
37528	LLLLTVLTV	Mucin 1, cell surface associated	23	6.35
1212707	FKFNEENY	Ataxin-2-like protein	15	4.14
167942	ITAGAHRLW	Stearoyl-CoA desaturase	15	4.14
472635	VLPLTVAEV	Mesothelin	12	3.31
1722079	AMPFATPMEA	Cancer/testis antigen 1 (NY-ESO-1)	11	3.04
62543	SYFPEITHI	Tyrosine-protein kinase JAK1	11	3.04
1310121	ILDTAGHEEY	neoantigen: GTP-binding protein GEM	10	2.76
1083942	CMLGTYTQDF	Calcitonin	9	2.49
45552	NQFPGFKEV	U2 small nuclear ribonucleoprotein B	8	2.21
2232678	GAARASGPGGGAPRG	Cancer/testis antigen 1 (NY-ESO-1)	7	1.93
2189284	RTFVLRVRAQDPPPE	Telomerase reverse transcriptase	11	3.00
2232660	EFYLAMPFATPMEAE	Cancer/testis antigen 1 (NY-ESO-1)	5	1.38
1355458	GVGSPYVSRLLGICL	Erb-b2 receptor tyrosine kinase 2	4	1.09
14375	ETFNTPAMY	Actin, cytoplasmic 1	3	0.82
137754	GVLVGVALI	Carcinoembryonic antigen-related cell adhesion molecule 5	3	0.82
434121	LHLGYLPNQL	ATP-dependent RNA helicase DDX1	2	0.54
47777	PGVLLKEFTVSGNILTIRLTAADHR	Cancer/testis antigen 1 (NY-ESO-1)	2	0.54
196522	HPPPPPPPP	Heterogeneous nuclear ribonucleoprotein L	1	0.27
223378	LRVAPEEHPVL	Actin, cytoplasmic 1 (NY-ESO-1)	1	0.27

Supplemental Table S7. Cancer peptides predicted to bind to T cell receptors detected in the study and the number of TCR-peptide matches
